# Physiology and anatomy of neurons in the medial superior olive of the mouse

**DOI:** 10.1152/jn.00523.2016

**Published:** 2016-09-21

**Authors:** Matthew J. Fischl, R. Michael Burger, Myriam Schmidt-Pauly, Olga Alexandrova, James L. Sinclair, Benedikt Grothe, Ian D. Forsythe, Conny Kopp-Scheinpflug

**Affiliations:** ^1^Division of Neurobiology, Department of Biology II, Ludwig Maximilian University Munich, Planegg-Martinsried, Germany;; ^2^Department of Biological Sciences, Lehigh University, Bethlehem, Pennsylvania; and; ^3^Department of Neuroscience, Psychology, and Behaviour, University of Leicester, Leicester, United Kingdom

**Keywords:** medial superior olive, auditory brain stem, K_v_1, K_v_3, mouse

## Abstract

In mammals with good low-frequency hearing, the medial superior olive (MSO) computes sound location by comparing differences in the arrival time of a sound at each ear, called interaural time disparities (ITDs). Low-frequency sounds are not reflected by the head, and therefore level differences and spectral cues are minimal or absent, leaving ITDs as the only cue for sound localization. Although mammals with high-frequency hearing and small heads (e.g., bats, mice) barely experience ITDs, the MSO is still present in these animals. Yet, aside from studies in specialized bats, in which the MSO appears to serve functions other than ITD processing, it has not been studied in small mammals that do not hear low frequencies. Here we describe neurons in the mouse brain stem that share prominent anatomical, morphological, and physiological properties with the MSO in species known to use ITDs for sound localization. However, these neurons also deviate in some important aspects from the typical MSO, including a less refined arrangement of cell bodies, dendrites, and synaptic inputs. In vitro, the vast majority of neurons exhibited a single, onset action potential in response to suprathreshold depolarization. This spiking pattern is typical of MSO neurons in other species and is generated from a complement of K_v_1, K_v_3, and *I*_H_ currents. In vivo, mouse MSO neurons show bilateral excitatory and inhibitory tuning as well as an improvement in temporal acuity of spiking during bilateral acoustic stimulation. The combination of classical MSO features like those observed in gerbils with more unique features similar to those observed in bats and opossums make the mouse MSO an interesting model for exploiting genetic tools to test hypotheses about the molecular mechanisms and evolution of ITD processing.

## Abstract (precis)

*The medial superior olive (MSO) is an important brain center that computes sound location by comparing small differences in arrival time at the two ears. The MSO is investigated for its specializations for processing with extreme temporal precision. In mice, the MSO is small and difficult to study; thus utilization of genetic methods in MSO studies has been lacking. We present the first comprehensive study of the murine MSO and show that most features are consistent with more heavily investigated species*.

## NEW & NOTEWORTHY

*The medial superior olive (MSO) is an important brain center that computes sound location by comparing small differences in arrival time at the two ears. The MSO is investigated for its specializations for processing with extreme temporal precision. In mice, the MSO is small and difficult to study; thus utilization of genetic methods in MSO studies has been lacking. We present the first comprehensive study of the murine MSO and show that most features are consistent with more heavily investigated species*.

a fundamental feature of all sensory processing is the determination of stimulus location. In the visual and somatosensory systems, stimulus location is encoded at the sensory surface. In the case of the auditory system, sound locations are not represented at the level of the ear but rather must be computed centrally in the brain stem.

Most mammals can hear sound frequencies of up to several tens of kilohertz, and they experience ample interaural level differences (ILDs) and spectral cues because of the strong shadowing effects of the head. Therefore it is not surprising that all terrestrial mammals can process ILDs (Grothe and Pecka 2014). However, for low frequencies these ILDs become small or even absent, simply because longer wavelengths are poorly reflected by the head. At these frequencies, interaural time differences (ITDs) in the submillisecond range become a useful localization cue. The extent to which an animal can utilize ITDs depends on the animal's auditory frequency range and its head size. Maximum detectible ITDs are limited by interaural distance; thus small-headed animals have a limited range of available ITDs. Traditionally, it had been postulated that the medial superior olive (MSO) in these exclusively high-frequency-hearing mammals is therefore of limited relevance (Masterton et al. 1967). Surprisingly, the MSO persists as an identifiable structure in nearly every small mammal investigated to date (gerbil: Couchman et al. 2012; Fischl et al. 2012; Grothe and Sanes 1993; Kuwabara and Zook 1992; Magnusson et al. 2005; Scott et al. 2005; guinea pig: Dietz et al. 2014; Saint Marie and Baker 1990; Smith 1995; mouse: Kopp-Scheinpflug et al. 2015; Kuwabara and Zook 1992; Ollo and Schwartz 1979; Yassin et al. 2014; Zettel et al. 2007; rat: Koch et al. 2004; Lohrke et al. 2005; Smith et al. 2000; and bat: Grothe and Pecka 2014).

MSO neurons have been studied extensively in vitro in gerbils and guinea pigs and in vivo in primarily cats and gerbils, all mammals with audiograms that extend into the low frequency range. Across species, MSO neurons are characterized by highly stereotyped anatomical and biophysical features such as *1*) their bipolar morphology (Ollo and Schwartz 1979; Rautenberg et al. 2009; Scott et al. 2005; Smith 1995; Smith et al. 2000), *2*) bilateral excitatory input from the bushy cells in the ventral cochlear nucleus (Cant and Casseday 1986; Goldberg and Brown 1969; Smith et al. 1993; Yin and Chan 1990), *3*) bilateral inhibitory inputs from the medial and lateral nuclei of the trapezoid body (Cant and Hyson 1992; Clark 1969; Grothe and Sanes 1994; Kuwabara and Zook 1992; Perkins 1973), *4*) the generation of temporally precise action potentials upon coincident excitatory input with additional inhibitory inputs helping to sharpen the ITD computation (Brand et al. 2002; Couchman et al. 2010; Fischl et al. 2012; Grothe and Sanes 1994; Kuwabara and Zook 1992; Myoga et al. 2014; Pecka et al. 2008; Stange et al. 2013; Yin and Chan 1990), *5*) strong expression of low-voltage-activated potassium (K_v_1) currents and hyperpolarization-activated nonspecific cation currents (*I*_H_) creating a low input resistance (*R*_input_) and a fast membrane time constant (τ) that refines the temporal window for coincidence detection (Baumann et al. 2013; Khurana et al. 2011, 2012; Koch et al. 2004; Mathews et al. 2010), and *6*) sending their major output projections to the neurons in the ipsilateral inferior colliculus (Adams 1979; Oliver et al. 1995).

Here we report biophysical, morphological, synaptic, and biochemical properties of mouse MSO neurons. In general, they share many, although not all, of the hallmark characteristics of MSO neurons in low-frequency-hearing mammals. Comparable biophysical and synaptic properties between MSO neurons in mouse and other mammals provide a missing link between the extensive biophysical data of gerbil MSO and in vivo data from the high-frequency-hearing bat MSO. These data support the feasibility of applying powerful genetic tools available in the mouse model to future investigation of MSO function.

## METHODS

All procedures were approved by the Bavarian district government according to European Communities Council Directives.

### 

#### In vitro electrophysiology.

Mice of the C57BL/6 background strain at postnatal days 11–26 (P11–P26) were anesthetized with isoflurane and rapidly decapitated. The brain stem containing auditory nuclei was removed, mounted, and submerged in oxygenated artificial cerebrospinal fluid (ACSF) bubbled with 95% O_2_-5% CO_2_ (containing in mM: 125 NaCl, 2.5 KCl, 10 glucose, 1.25 NaH_2_PO_4_, 26 NaHCO_3_, 2 CaCl_2_, 1 MgCl_2_, 0.5 ascorbic acid, 3 *myo*-inositol, 2 pyruvic acid) (Sigma-Aldrich, St. Louis, MO) at 22°C. The brain stem was mounted on a metal block with cyanoacrylate glue for coronal sectioning on the stage of a vibrating microtome (VT1200s; Leica). Coronal sections (200 μm) containing the MSO were collected and submerged in a 37°C incubation chamber of continuously oxygenated ACSF and incubated for 30–60 min. Slices were then maintained in oxygenated ACSF at room temperature until being used for recording.

Brain stem slices were placed in a recording chamber, and neurons were visualized with an Olympus microscope (BX51WI) using infrared differential interference contrast optics. Video images were captured with a CCD camera coupled to a video monitor. The recording chamber was continuously perfused with ACSF at a rate of 2–4 ml/min. An in-line feedback temperature controller and heated stage were used to maintain chamber temperature at 36 ± 1°C (TC344B; Warner Instruments, Hamden, CT).

#### Patch-clamp recordings.

Principal MSO neurons were identified based on several criteria: *1*) their location relative to reliable landmarks in the slice, *2*) their characteristic bipolar morphology (assessed post hoc, see below), *3*) low *R*_input_ upon achieving a whole cell configuration (Scott et al. 2005), and *4*) characteristic spiking pattern in response to depolarizing current steps in current clamp (Scott et al. 2005; Smith 1995). For evoked postsynaptic current (PSC) recordings, a concentric bipolar electrode with tungsten core (FHC) was lowered to the tissue surface with a micromanipulator (Luigs & Neumann) and placed in a position either medial to the MSO near the medial nucleus of the trapezoid body (MNTB) or along the lateral margins of the slice near the lateral superior olive (LSO). Excitatory fibers putatively from anterior ventral cochlear nucleus as well as inhibitory fibers from the ipsilateral MNTB were then stimulated.

Patch pipettes were pulled from borosilicate glass capillary tubes (GC150F-7.5, OD 1.5 mm; Harvard Apparatus, Edenbridge, UK) with a multiple-stage puller (DMZ puller; Zeitz, Munich, Germany) and back-filled with internal solution [containing in mM: 126 K-gluconate, 4 KCl, 1 MgCl_2_, 40 HEPES, 5 EGTA, Na_2_ phosphocreatine (pH adjusted to 7.2 with KOH)] (Sigma-Aldrich) used for both current- and voltage-clamp recordings. Final resistances were 3–4 MΩ. Whole cell access resistances were <10 MΩ. All voltages were off-line corrected for a 14-mV liquid junction potential. In voltage clamp, series resistance was compensated at 60–80%. For some PSC recordings, 5 mM QX314 was added to the internal solution to prevent antidromic action potentials. Membrane voltage was clamped at −60 mV with a HEKA EPC-10 amplifier and recorded with Heka Patchmaster software, sampling at 50 kHz and filtering at 10 kHz. Excitatory PSCs (EPSCs) were evoked with brief voltage shocks to synaptic input fiber tracts in the slice (Pulse Stimulator AM-2100). Stimulus magnitude (range 10–90 V) was gradually increased until PSC amplitudes stabilized at their maximum amplitude. Excitatory currents were recorded in ACSF containing the GABA_A_ receptor blocker SR95531 (20 μM) and the glycine receptor blocker strychnine (1 μM). To isolate inhibitory inputs, ACSF containing 6,7-dinitroquinoxaline-2,3-dione (DNQX; 10 μM) and d-2-amino-5-phosphonopentanoic acid (d-AP5; 50 μM) was used to block AMPA and NMDA glutamate receptors, respectively. Membrane voltage was clamped at −40 mV for inhibitory postsynaptic current (IPSC) data collection. Paired-pulse ratios (PPRs) were measured in response to two consecutive stimuli with a delay of 50 ms. Paired-pulse stimuli were presented 20 times at a rate of 0.5 Hz. Miniature PSCs were detected by creating a template for each cell in Clampfit 10.2 (Molecular Devices). Traces were then verified, and false positives were excluded. Pharmacological compounds were obtained from Sigma-Aldrich unless otherwise indicated.

#### Post hoc analyses.

Patchmaster files were converted with a custom MATLAB script (MathWorks; Prof. Achim Klug, University of Colorado School of Medicine, Denver, CO) and formatted to be compatible for analysis with Clampfit. Intrinsic properties as well as PSC amplitudes and kinetics were analyzed with Clampfit software. Statistical analyses of the data were performed with SigmaStat/SigmaPlot (SPSS Science, Chicago, IL). Results are reported as means ± SE, with *n* being the number of neurons recorded from at least three different animals. Statistical comparisons between different data sets were made with unpaired Student's *t*-test, or Mann-Whitney rank-sum test if nonnormally distributed, while before and after comparisons were made with paired Student's *t*-test. Differences were considered statistically significant at *P* < 0.05. Analysis of variance (ANOVA) was used when test groups numbered more than two. Existence of correlation between variables was tested with Pearson product moment correlation.

#### Anatomical verification.

In many cases, 0.4% biocytin was added to the internal solution to label the neurons according to the protocol of Scott et al. (2005). Slices were fixed in ∼0.5 ml of phosphate-buffered saline solution (PBS) containing 4% paraformaldehyde (PFA) after recording sessions in individual wells of a 24-well culture plate. Fixed slices were washed in PBS after fixation periods of 24 h to 2 wk. Membranes were permeabilized with overnight rinse in 3% Triton X-100 in PBS. Tissue was washed in a blocking solution of PBS containing 3% Triton X-100, 1% bovine serum albumin (BSA), and 0.1% saponin. Microtubule-associated protein 2 (MAP2) protein was immunolabeled with a MAP2 antibody (1:1,000 in blocking solution; Neuromics CH22103, Edina, MN). Biocytin was labeled with streptavidin conjugated to Cy3 (1:500 in blocking solution; Dianova no. 016-160-084). Counterstaining was achieved with Ch-A647 (1:1,000; Dianova) and NeuroTrace green (1:100 in blocking solution; Invitrogen N21480), followed by repeated washes in blocking solution (15 min), and then 3 × 10 min in PBS. Slices were mounted on gelatin-coated slides with fluorescent mounting medium (Vectashield H-1000; Vector Labs) before confocal imaging (see below).

#### In vivo physiology.

Animals were anesthetized with a subcutaneous injection of 0.01 ml/g MMF (0.5 mg/kg body wt medetomidine, 5.0 mg/kg body wt midazolam, and 0.05 mg/kg body wt fentanyl) and were placed on a temperature-controlled heating pad (ATC1000; WPI) in a soundproof chamber (Industrial Acoustics). Depth of anesthesia was measured with the toe pinch reflex, and animals responding were given supplemental MMF at one-third the initial dose. The mice were then stabilized in a custom stereotaxic device. An incision was made at the top of the skull, and a head post was fixed to the skull with dental cement. A craniotomy was performed above the cerebellum to access the auditory brain stem. A ground electrode was placed in the muscle at the base of the neck. Glass microelectrodes were pulled from glass capillary tubes (GC150F-7.5; Harvard Apparatus) so that the resistance was 5–20 MΩ when filled with 3 M KCl solution or 2 M potassium acetate with 2.5% biocytin. Signals were amplified (Neuroprobe Amplifier model 1600; A-M Systems), filtered (300-3,000 Hz; TDT PC1), and recorded (∼50 kHz sampling rate) with an RZ6 processor (TDT). SPIKE software (Brandon Warren, V. M. Bloedel Hearing Research Center, University of Washington) was used to calibrate the speakers (MF1; TDT), generate stimuli, and record action potentials. Stimuli consisted of pure tones (50- to 100-ms duration, 5-ms rise/fall time) at varying intensity (0–90 dB SPL) and were presented through hollow ear bars connected to the speakers with Tygon tubing. Spike sorting and data analysis were performed off-line with custom MATLAB programs. At the end of the experiment, biocytin (2.5%) was deposited at the final penetration with the current injection mode of the amplifier (Neuroprobe model 1600; A-M Systems; +0.5 μA, 1–2 min) Thirty minutes was allowed for cellular uptake before the animal was perfused and the tissue was processed for biocytin fluorescence as described above. Recording sites were determined by using the biocytin deposition as a reference for stereotaxic reconstruction.

#### Retrograde tracing.

For retrograde tracing experiments mice were anesthetized with MMF (see above). A solution (2 μl) containing 5% Fluoro-Gold was pressure-injected into the inferior colliculus with a stereotaxic device (Neurostar). At the end of the procedure mice received a subcutaneous antidote injection of 2.5 mg/kg body wt atipamezole, 0.5 mg/kg body wt flumazenil, and 1.2 mg/kg body wt naloxone. After a 5- to 7-day recovery period, animals were perfused (see below) and brain sections taken for subsequent fluorescence microscopy.

#### Immunohistochemistry.

C57BL/6 mice (aged 2–3 mo) were euthanized with an overdose of pentobarbital and perfusion-fixed with 4% PFA in PBS intracardially. After overnight postfixation in 4% PFA, brain stems were sectioned coronally or horizontally, creating sections 50–100 μm thick with a vibratome (Leica 120S). After rinsing in PBS, sections were transferred to a blocking solution containing 1% BSA, 0.5% Triton X-100, and 0.1% saponin in PBS. The floating sections were then incubated for 48 h at 4°C in blocking solution containing primary antibodies (Table 1). Sections were washed three times in PBS for 15 min and incubated with secondary antibodies overnight at 4°C (Table 2). Sections were washed in PBS, mounted on slides, and coverslipped with Vectashield mounting medium.

**Table 1.** Primary antibodies used for immunocytochemistry

Primary Antibody	Antigene	Supplier	Catalog No.	Host	Dilution
ChAT	Human placental enzyme (mol wt 70,000/74,000)	Millipore	AB144P	Goat	1:400
GLYT2	Recombinant protein of rat (aa 1–229)	SySy	272003	Rabbit	1:1,000
MAP2	Microtubule-associated protein 2	Neuromics	CH22103	Chicken	1:1,000
NeuN	Purified cell nuclei from mouse brain	Millipore	MAB377	Mouse (IgG1)	1:200
VGLUT1	Purified recombinant protein of rat (aa 456–560)	SySy	135304	Guinea pig	1:2,000

**Table 2.** Secondary antibodies used for immunocytochemistry

Secondary Antibody	Conjugate	Supplier	Catalog No.	Host	Dilution
Anti-chicken	Cy3	Dianova	703-166-155	Donkey	1:200
Anti-chicken	AMCA	Dianova	703-156-155	Donkey	1:100
Anti-chicken	Alexa 488	Dianova	703-546-155	Donkey	1:300
Anti-goat	Cy5	Abcam	Ab6566	Donkey	1:100
Anti-guinea pig	Cy3	Dianova	706-166-148	Donkey	1:300
Anti-guinea pig	Alexa 488	Molecular Probes	A11073	Goat	1:200
Anti-mouse	Alexa 488	Dianova	715-545-150	Donkey	1:200
Anti-rabbit	Alexa 633	Molecular Probes	A21071	Goat	1:200

Antibodies directed against vesicular glutamate transporter type 1 (VGLUT1) and glycine transporter 2 (GLYT2) are shown to recognize expected bands at ∼60 kDa and ∼100 kDa, respectively (Western blot analysis; data sheet from Synaptic Systems). For choline acetyltransferase (ChAT), a polyclonal antibody (AB144P; Millipore) was used that recognizes a single expected band at ∼70 kDa (Western Blot analysis; Kaiser et al. 2011). Antibodies directed against MAP2 are shown to detect MAP2a and -b variants at ∼280 kDa and -c variant at 70 kDa in Western blot analysis (data sheet and personal communication from Neuromics). This was confirmed in immunocytochemical control experiments carried out in a previous investigation (Rautenberg et al. 2009). We routinely carry out control experiments in which we test our secondary antibodies by omitting the primary antibody. Application of exclusively secondary antibodies showed no staining pattern (data not shown). To confirm the specificity of double- and triple immunolabeling experiments, we compared double-/triple-stained brain sections with single-stained sections. We showed that application of several primary antibodies simultaneously did not change the resulting pattern of single-antigen distribution (data not shown). We also tested the specificity of immunolabeling by omitting the primary antibodies raised against the second/third antigen in our scheme and applying only corresponding secondary antibodies. In this case second/third secondary antibodies demonstrated no staining pattern.

#### Confocal microscopy.

Confocal optical sections were acquired with a Leica TCS SP5-2 confocal laser-scanning microscope (Leica Microsystems, Mannheim, Germany) equipped with HCX PL APO CS 20X/NA0.7, HCX PL APO Lambda Blue X63/numerical aperture 1.4 oil immersion objectives. Fluorochromes were visualized with excitation wavelengths of 405 nm (emission filter 410–450 nm) for Fluoro-Gold and AMCA, 488 nm (emission filter 510–540 nm) for Alexa 488, 561 nm (emission filter 565–585 nm) for Cy3, 594 nm (emission filter 605–625 nm) for DyLight 594, and 633 nm (emission filter 640–760 nm) for Alexa 633. For each optical section the images were collected sequentially for two to five fluorochromes. Stacks of 8-bit grayscale images were obtained with axial distances of 290 nm between optical sections and pixel sizes depending on the selected zoom factor and objective. To improve the signal-to-noise ratio, images were averaged from three successive scans. After stack acquisition, chromatic aberration-induced Z shift between color channels was corrected for with a custom plug-in written by Dr. Boris Joffe (Ludwig Maximilian University Munich, Biocenter). RGB stacks, montages of RGB optical sections, and maximum-intensity projections were assembled with ImageJ 1.37k plug-ins and Adobe Photoshop 8.0.1 (Adobe Systems, San Jose, CA) software.

#### Single-cell three-dimensional reconstructions.

With the ImageJ 1.37k paint-brush tool, single cells were manually labeled by following successively through each optical section of the confocal image stack. Subsequently, the neighboring structures were digitally deleted. We refer to this method as digital extraction. Three-dimensional (3D) reconstructions were produced with the Image J 1.37 plug-in ImageJ 3D Viewer.

## RESULTS

### 

#### VGLUT1 enables identification of MSO in mouse between MNTB and LSO.

**Fig. 1.**
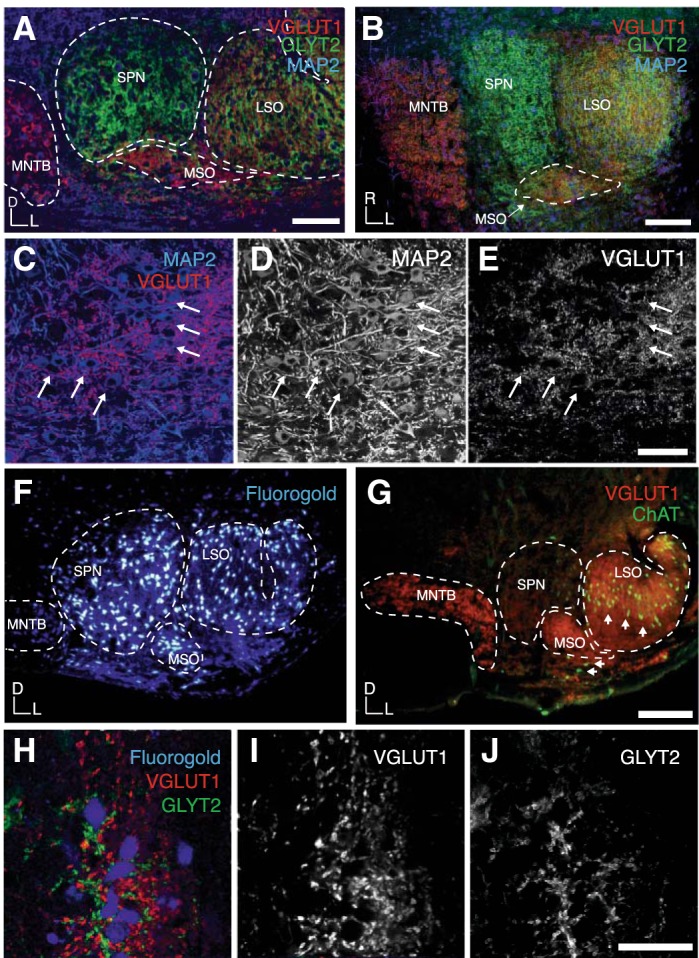
Location and histological characterization of the MSO in mice. *A*: coronal section through the SOC with immunostaining for GLYT2 and VGLUT1. The SPN and MNTB are singly labeled for GLYT2 and VGLUT1, respectively, while the LSO and MSO appear immunopositive for both. Dashed line depicts the overall MSO outline including the dendrites. *B*: horizontal section through the SOC with the MSO highlighted by the dashed line. *C*: merged image of MAP2-labeled MSO neurons (arrows) and their VGLUT1-positive inputs. *D*: MAP2 stain of image in *C*. *E*: VGLUT1 stain of image in *C*. *F*: retrogradely labeled neurons in the SPN, LSO, and MSO after Fluoro-Gold injections into the ipsilateral inferior colliculus. *G*: VGLUT labeling of MSO and MNTB is distinct from ChAT labeling of the MOC and LOC neurons (arrows). *H*: MSO neurons, retrogradely labeled by Fluoro-Gold, are colabeled for VGLUT1 and GLYT2. *I*: VGLUT1 stain of image in *H*. *J*: GLYT2 stain of image in *H*. Scale bars: 100 μm (*A*, *C–E*), 200 μm (*B*, *G*), 40 μm (*H–J*).

The presence and location of the MSO within the mouse superior olivary complex (SOC) could not be clearly identified with Nissl staining (data not shown). However, specific combinations of immunohistochemical markers for excitatory and inhibitory presynaptic proteins with known expression patterns within the SOC allowed us to clearly differentiate MSO from its neighboring nuclei in both coronal (Fig. 1*A*) and horizontal (Fig. 1*B*) sections. Antibodies against VGLUT1 (Fig. 1, *A* and *B*) were applied to visualize the excitatory inputs, while glycinergic inhibitory inputs were identified by using antibodies against the neuronal GLYT2 (Fig. 1, *A* and *B*). The MNTB receives VGLUT1-positive excitatory inputs and little glycinergic input. The neurons of the superior paraolivary nucleus (SPN) are located dorsomedially to the MSO. Our staining revealed that compared with MNTB, LSO, or MSO neurons, SPN neurons receive few VGLUT1-positive excitatory inputs, a result similar to that previously reported in rats (Blaesse et al. 2005). This weaker VGLUT1 labeling made it easier to delineate SPN from MSO. Here, we also observed strong labeling for the neuronal GLYT2 (Fig. 1, *A* and *B*). Neurons in MSO and LSO receive both excitatory (VGLUT1 positive) and inhibitory (GLYT2 positive) input (Fig. 1, *A* and *B*). Therefore in overview images with low resolution acquired with an epifluorescence microscope or in maximum-projection confocal images MNTB and SPN can be seen as “red” and “green” nuclei, respectively, while LSO and MSO have a yellowish appearance (result of overlay of red and green false colors used for depicting VGLUT1 and GlyT2 signals in RGB mode) (Fig. 1, *A* and *B*). The excitatory VGLUT1-positive input preferentially, although not exclusively, targets the dendrites of the MSO neurons (Fig. 1, *C–E*), giving the MSO an elongated shape (Fig. 1) with the MAP2-positive somata in the center (Fig. 1*A*). The location of the MSO within the mouse SOC was further confirmed by injections of the tracer Fluoro-Gold into the inferior colliculus, resulting in retrograde labeling of SPN, MSO, and LSO, but not MNTB, neurons in the ipsilateral SOC (Fig. 1*F*). In four of five mice in which we combined retrograde Fluoro-Gold labeling with immunohistochemistry, MSO neurons were successfully labeled with the tracer. All Fluoro-Gold-positive MSO cells showed positive staining for excitatory (VGLUT1) and inhibitory (GLYT2) inputs (Fig. 1, *H–J*).

Clusters of cholinergic neurons reside medially to the LSO in close proximity to the MSO and constitute the origin of the medial olivocochlear pathway (MOC cells). To rule out that putative MSO neurons were MOC neurons, we immunolabeled for ChAT, a marker of cholinergic neurons (Fig. 1*G*). No ChAT-positive neurons were found in the circular VGLUT1-positive area identified as MSO.

The borders of the MSO were defined in coronal or horizontal sections of three mice with a combination of MAP2/NeuN/VGLUT1/GLYT2 immunolabeling. In coronal slices the MSO appeared generally as an ellipse that was ∼150 μm in the mediolateral dimension and 100 μm in the dorsoventral dimension. The MSO could be delineated with this staining method in ∼8–10 coronal sections (50 μm thick), suggesting a rostrocaudal dimension between 400 and 500 μm. VGLUT1 labeling was used to draw an outline of the MSO including its dendritic area in a ×10 magnification. The number of neurons based on their MAP2 and NeuN labeling inside this outline was then counted for each section with confocal stacks and ImageJ plug-ins. A conservative measure of only neurons within the VGLUT1-positive area constituted 247 ± 17 MSO neurons (*n* = 4).

#### Passive and active intrinsic properties of mouse MSO neurons.

**Fig. 2.**
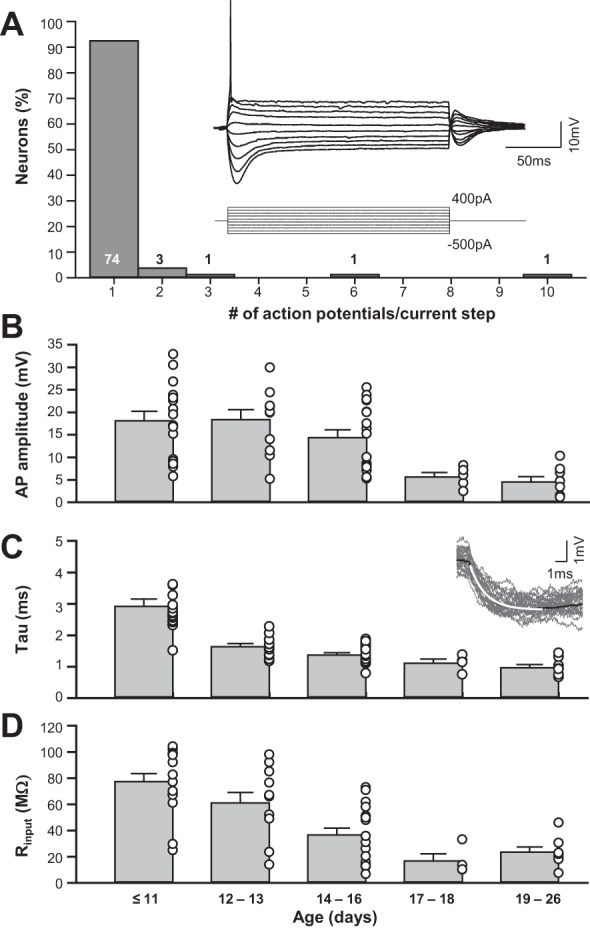
Single-spike firing pattern and passive properties of MSO neurons in mice resemble those of other rodents. *A*: distribution of spike firing patterns in mouse MSO neurons. *Inset* shows characteristic voltage response to prolonged current injections. *B*: absolute action potential (AP) amplitudes decrease with increasing postnatal age. *C*: membrane time constants acquired by fitting a single-exponential decay function to a voltage response following small current injections decrease with increasing postnatal age. *D*: input resistance of MSO neurons also decreases with increasing postnatal age.

MSO neurons in gerbil and rat exhibit very low *R*_input_ because of high expression of low-voltage-gated K_v_ and *I*_H_ conductances (Khurana et al. 2011; Kopp-Scheinpflug et al. 2015; Scott et al. 2007; Smith et al. 2000). In mouse MSO whole cell current-clamp recordings, prolonged depolarizing current injection resulted in the generation of a single action potential in 92% (74/80) of neurons (Fig. 2*A*, *inset*). Injections of hyperpolarizing currents resulted in a rapidly activating depolarizing sag in membrane potential indicative of the strong *I*_H_ currents, typical of MSO neurons. Absolute amplitudes of single action potentials decreased from 18.11 ± 2.09 mV (*n* = 16) around hearing onset to 4.53 ± 1.18 mV (*n* = 8) at the end of the third postnatal week (Mann-Whitney rank sum test: *P* ≤ 0.001; Fig. 2*B*). The resting membrane potential of the MSO neurons was −61.04 ± 0.62 mV (*n* = 80) and did not differ over the age range that we sampled (P11–P26) [1-way repeated-measures ANOVA (RM-ANOVA): *P* = 0.437]. We calculated the membrane time constant (τ) and the *R*_input_ of these neurons by fitting a single-exponential function to the initial decay of the average (30 repetitions) response to a −10-pA hyperpolarizing current step (Fig. 2, *C* and *D*); τ accelerated from 2.91 ± 0.23 ms (*n* = 16) at P11 to 0.96 ± 0.10 ms (*n* = 8) by the end of the third postnatal week (Mann-Whitney rank sum test: *P* ≤ 0.001; Fig. 2*C*). It should be noted that the strongest acceleration appeared between P11 and P12/13, while the further decline between P12/13 and ≥P19 was no longer significant (1-way RM-ANOVA). In addition to the acceleration of τ, the neurons' *R*_input_ dropped from 77.36 ± 6.08 MΩ (*n* = 16) at P11 to 23.54 ± 9.26 MΩ (*n* = 8) at the end of the third postnatal week (Mann-Whitney rank sum test: *P* ≤ 0.001; Fig. 2*D*). No additional significant changes were observed after P16/17 (1-way RM-ANOVA).

#### Contribution of low- and high-voltage-activated K^+^ currents to action potential firing in MSO.

**Fig. 3.**
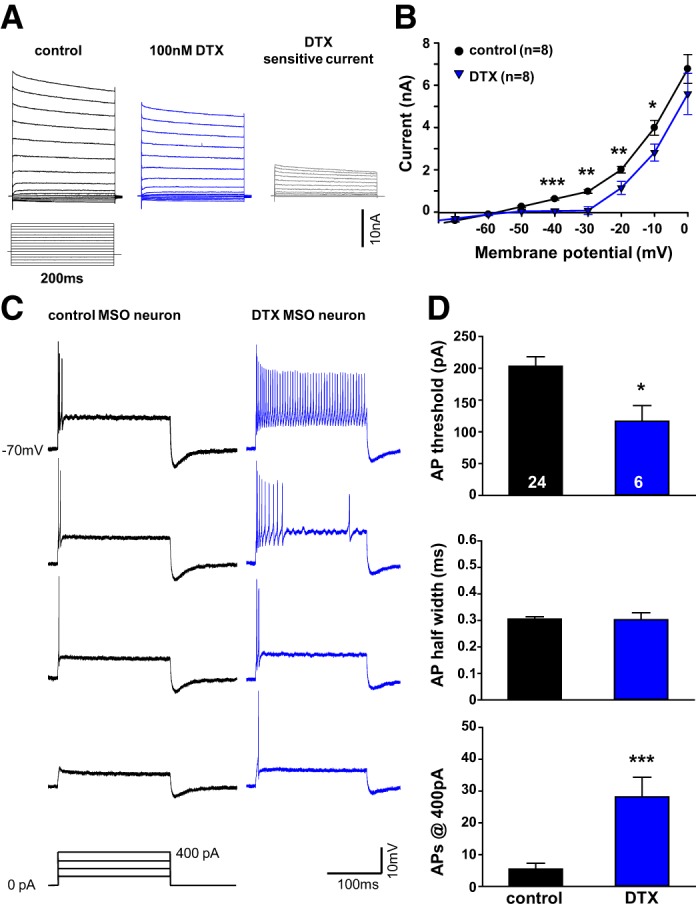
DTX-sensitive K_v_1 potassium currents determine MSO neurons' threshold and firing pattern. *A*: outward currents of an MSO neuron (P12) stepped to potentials between −110 mV and +50 mV for 200 ms in control (black) and DTX (blue). Gray traces show the subtracted, DTX-sensitive outward current. *B*: current-voltage functions show a significant reduction in the low-voltage-activated outward currents between −40 mV and −10 mV in P12–P13 mice. *C*: action potentials (APs) in response to depolarizing current injections of +100 pA to +400 pA for 200 ms in current-clamp mode for neuron shown in *A* before (black) and after (blue) DTX treatment. *D*: AP threshold decreases (*top*), AP half-width remains unchanged (*middle*), and AP number increases (*bottom*) during blockade of DTX-sensitive currents in P12–P19 mice. *P ≤ 0.01, **P ≤ 0.005, ***P ≤ 0.001.

The single action potential response to prolonged depolarization is a common feature of MSO neurons. Similar firing patterns in MNTB neurons have previously proven to be dependent on fast activating but slowly inactivating K_v_1 potassium currents that remain open for the duration of the depolarizing pulse and limit subsequent action potential firing (Barnes-Davies et al. 2004; Brew and Forsythe 1995; Johnston et al. 2010). Here, voltage-clamp recordings of outward currents in response to voltage steps between −110 and +50 mV in control ACSF and in 100 nM dendrotoxin (DTX) were performed to quantify the K_v_1 currents in MSO neurons. After DTX application, the K_v_1-mediated current dropped significantly between −40 mV and −10 mV (Fig. 3, *A* and *B*). Despite the small DTX-sensitive current amplitude of 0.89 ± 0.14 nA at −40 mV (*n* = 8), the K_v_1 current had profound effects on the excitability of the MSO neurons. After DTX application the current required to generate an action potential dropped significantly from 203 ± 15 pA (*n* = 24) to 115 ± 30 pA (*n* = 6; Mann-Whitney rank sum test: *P* = 0.026; Fig. 3, *C* and *D*). While under control conditions the number of action potentials was restricted to 5.46 ± 1.8 (*n* = 24) even with current injections well above threshold (+400 pA), during DTX application the number of action potentials increased sharply to 28.17 ± 6.17/current step (*n* = 6, Mann-Whitney rank sum test: *P* ≤ 0.002; Fig. 3, *C* and *D*). No change in action potential half-width was observed after DTX application, suggesting that K_v_1 currents did not contribute to the repolarization of action potentials (Fig. 3*D*).

**Fig. 4.**
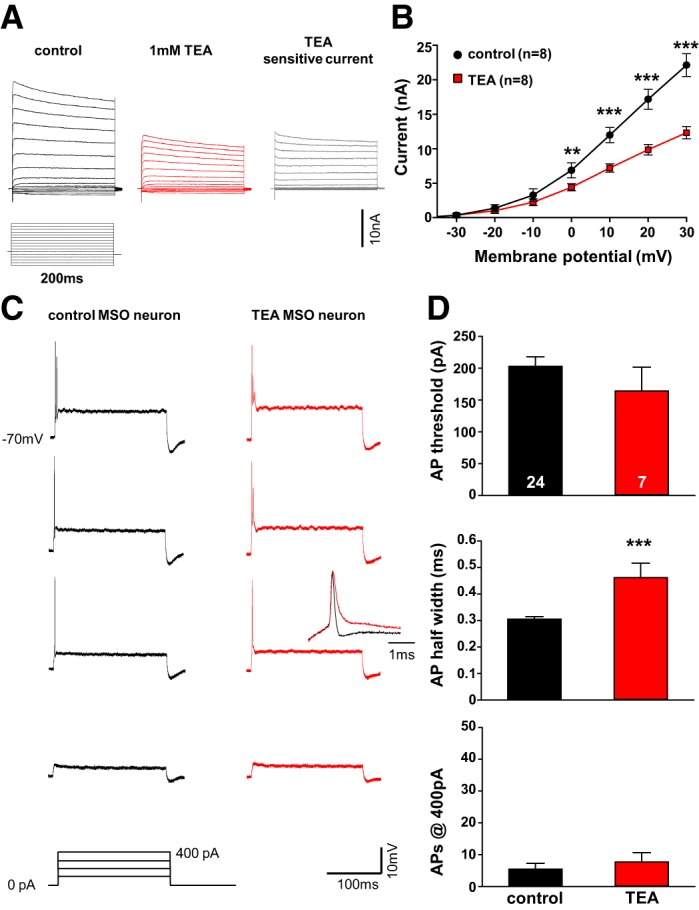
TEA-sensitive K_v_3 potassium currents have a large contribution to MSO neurons' half-width. *A*: outward currents of an MSO neuron (P12) stepped to potentials between −110 mV and +50 mV for 200 ms in control conditions (black) and in the presence of 1 mM TEA (red). Gray traces show the subtracted, TEA-sensitive outward current. *B*: current-voltage functions show a significant reduction in the high-voltage-activated outward currents between −10 mV and +30 mV in P12–P13 mice. *C*: action potentials (APs) in response to depolarizing current injections of +100 pA to +400 pA for 200 ms in current-clamp mode for neuron shown in *A* before (black) and after (red) TEA treatment. *D*: AP half-width increases (*middle*), while AP threshold (*top*) and AP number (*bottom*) remain unaltered during blockade of TEA-sensitive currents in P12-P19 mice. **P < 0.005, ***P < 0.001.

High-voltage-activated potassium channels of the K_v_3 family are strongly expressed in fast-spiking neurons of the auditory system, which often exhibit short refractory periods. K_v_3 channels open at voltages close to the action potential peak and are therefore a major contributor to repolarization (Johnston et al. 2010). Voltage-clamp recordings of MSO neurons in control ACSF revealed large outward currents of 22.13 ± 1.65 nA (*n* = 8) evoked by step depolarizations up to +30 mV (Fig. 4*A*). Application of the K_v_3 channel blocker TEA (1 mM) caused a significant reduction of the outward current at potentials equal to or greater than −10 mV. A TEA-sensitive K_v_3 component measuring 12.32 ± 0.89 nA (*n* = 8) in response to +30-mV steps was observed (paired *t*-test, *P* ≤ 0.001; Fig. 4, *A* and *B*). The functional role of K_v_3 current in MSO neurons was then studied in current-clamp recordings. Prolonged depolarizing current injections of +50 to +400 pA revealed the typical single action potential firing pattern at and above threshold. Application of 1 mM TEA did not affect the threshold current or the number of action potentials evoked at or above threshold (Fig. 4, *C* and *D*). However, action potential half-width broadened significantly from 0.3 ± 0.01 ms (*n* = 24) in control to 0.46 ± 0.05 ms (*n* = 7) during K_v_3 antagonist application, revealing the role of this channel in membrane repolarization during spiking (Mann-Whitney rank sum test: *P* ≤ 0.001; Fig. 4, *C*, *inset*, and *D*).

#### Mouse MSO neurons generate fast AMPA currents in response to afferent fiber stimulation.

**Fig. 5.**
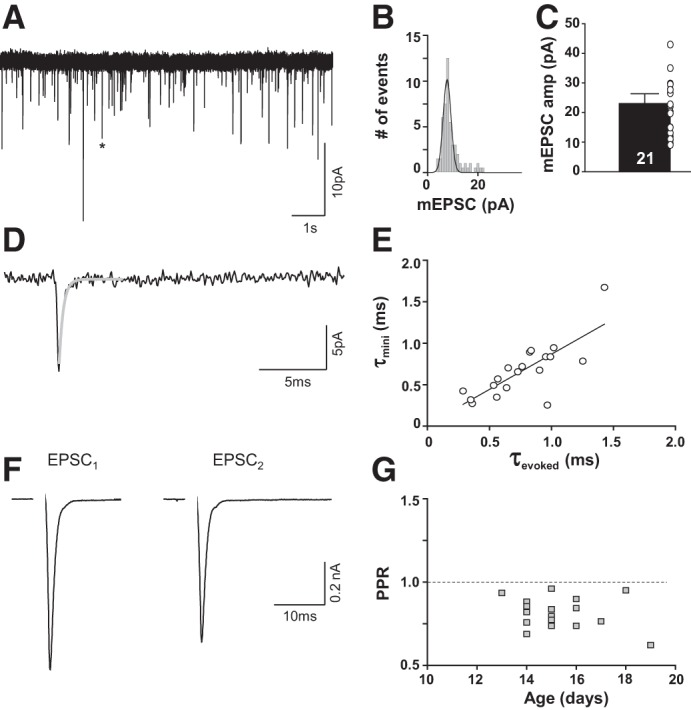
Excitatory synaptic inputs in mouse MSO neurons have fast decay time constants and are mediated by AMPA receptors. *A*: recording of mEPSCs. Asterisk indicates the specific mEPSC expanded in *D*. *B*: mEPSC peak distribution of cell in *A*. Solid line corresponds to a Gaussian fit. *C*: average mEPSC amplitudes. *D*: a single enlarged mEPSC marked by asterisk in *A* fitted with an exponential decay function (gray curve). *E*: decay time constant of the average mEPSC of each cell correlates to the corresponding evoked EPSCs. *F*: average trace (20 repetitions) of the paired-pulse response (50-ms interstimulus interval) of an MSO neuron showing depression. Stimulus artifacts were removed for better visualization. *G*: paired-pulse ratios (PPRs) from all individual cells plotted against postnatal age. If the PPR = 1 (dashed line) it implies no change in PSC size, indicating no facilitation or depression.

MSO neurons receive fast excitatory inputs from the spherical bushy cells in the cochlear nucleus. We evaluated responses to synaptic inputs in mouse MSO neurons by electrically stimulating the afferent fibers in the presence of the GABA and glycine blockers SR95531 (20 μM) and strychnine (1 μM), respectively. For each neuron, spontaneous miniature excitatory postsynaptic currents (mEPSCs) and evoked excitatory postsynaptic currents (eEPSCs) were recorded. For eEPSCs, both ipsi- and contralateral afferent fibers were stimulated. There were no significant differences between ipsilaterally vs. contralaterally stimulated eEPSCs for amplitude (ipsi: 331 ± 80 pA, *n* = 8; contra: 492 ± 105 pA, *n* = 12; Mann-Whitney rank sum test: *P* = 0.298) or decay time constant (ipsi: 0.59 ± 0.10 ms; contra: 0.83 ± 0.07 ms; *t*-test: *P* = 0.105), and data were therefore pooled. Afferent fiber stimulation with stimulus intensities of 66.3 ± 7.8 V reliably generated short-latency (1.58 ± 0.10 ms) eEPSCs in 20 of 22 neurons. At resting membrane voltages eEPSCs had mean amplitudes of 428 ± 72 pA and decay time constants of 0.74 ± 0.07 ms (*n* = 20). mEPSCs occurred with an average frequency of 10 ± 2 Hz and an amplitude of 23.09 ± 3.2 pA (*n* = 21; Fig. 5, *A–C*). The decay time constant of the mEPSCs (0.66 ± 0.07 ms; *n* = 21) was significantly correlated to the decay time constant of the maximum eEPSCs with a correlation coefficient of 0.772 (Pearson product moment correlation: *P* ≤ 0.001; Fig. 5, *D* and *E*). Decay times for both eEPSCs and mEPSCs were not significantly different from each other (*t*-test: *P* = 0.227).

Consistent short-term depression of excitatory transmission has been shown for the gerbil MSO (Couchman et al. 2010). We analyzed PPRs of maximum eEPSCs to evaluate whether the excitatory synaptic inputs to the mouse MSO are depressing or facilitating (Fig. 5*F*). eEPSCs in mouse MSO depressed with a PPR of 0.78 ± 0.03 (*n* = 19). PPR did not change significantly between P13 and P19 (Pearson product moment correlation: *P* > 0.05; Fig. 5*G*).

#### Mouse MSO neurons receive glycinergic input in response to stimulation of ipsilateral MNTB.

**Fig. 6.**
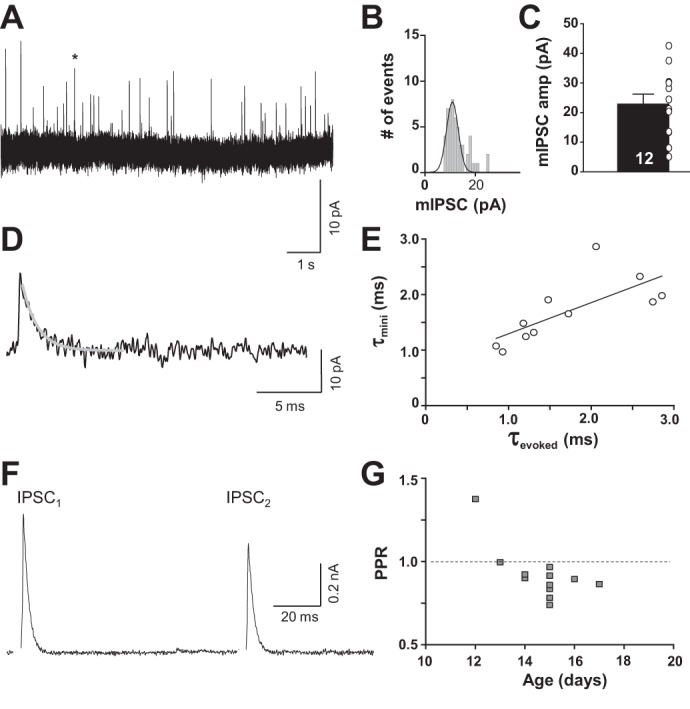
Inhibitory synaptic inputs in mouse MSO neurons are mediated by glycine receptors. *A*: recording of mIPSCs. Asterisk indicates the specific mIPSC expanded in *D*. *B*: mIPSC peak distribution of cell in *A*. Solid line corresponds to a Gaussian fit. *C*: average mIPSC amplitudes. *D*: a single enlarged mIPSC marked by asterisk in *A* fitted with an exponential decay function (gray curve). *E*: decay time constant of the average mIPSC of each cell correlates to the corresponding evoked IPSCs. *F*: average trace (20 repetitions) of the paired-pulse response of an MSO neuron showing depression. Stimulus artifacts were removed for better visualization. *G*: paired-pulse ratios (PPRs) from all individual cells plotted against postnatal age. A PPR of 1 (dashed line) indicates no short-term plasticity.

Glycinergic IPSCs were evoked by electrical stimulation of the ipsilateral MNTB and examined in the presence of the glutamate receptor blockers DNQX (10 μM) and d-AP5 (50 μM) and the GABA receptor blocker SR95531 (20 μM). For each neuron, spontaneous miniature IPSCs (mIPSCs) and evoked IPSCs (eIPSCs) were recorded. Electric stimulation of the ipsilateral MNTB generated eIPSCs in 14 of 15 MSO neurons with latencies of 1.52 ± 0.15 ms (*n* = 14) and mean amplitudes of 261 ± 46 pA (*n* = 14). mIPSCs occurred with an average frequency of 1.28 ± 0.22 Hz and amplitude of 22.87 ± 3.33 pA (*n* = 12; Fig. 6, *A–C*). The decay time constant of the mIPSCs (1.66 ± 0.16 ms; *n* = 12) was significantly correlated to the decay time constant of the maximum eIPSCs (1.68 ± 0.18 ms; *n* = 12) with a correlation coefficient of 0.726 (Pearson product moment correlation: *P* = 0.0114; Fig. 6, *D* and *E*). The decay times were not significantly different from each other (*t*-test: *P* = 0.923). Maximum eIPSCs were tested for short-term plasticity in a paired-pulse paradigm (Fig. 6*F*). Here we observed a PPR of 0.88 ± 0.02 (*n* = 11), indicating synaptic depression for postnatal ages ≥P14 (Fig. 6*G*).

#### Morphology of mouse MSO neurons.

**Fig. 7.**
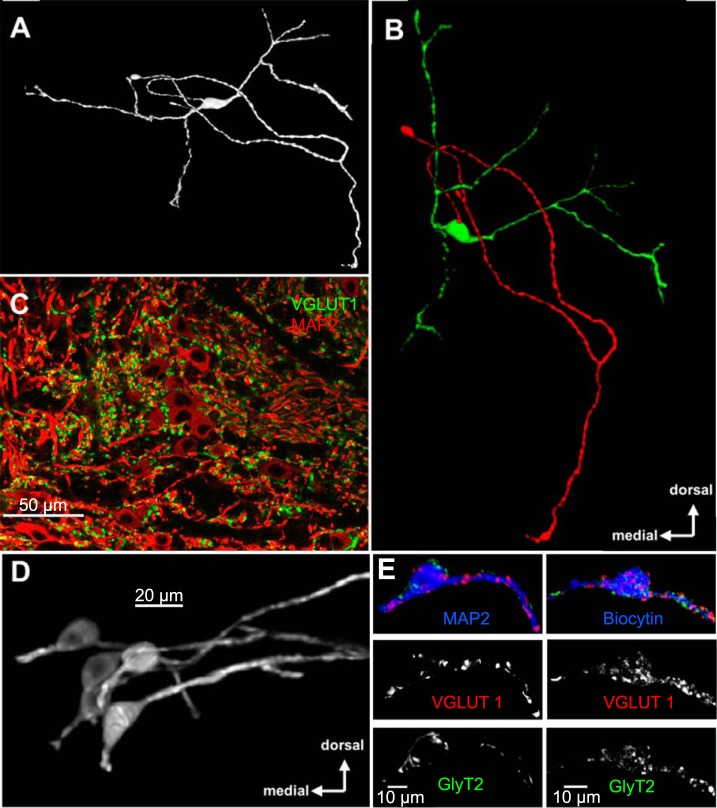
Bipolar MSO neurons do not show the typical planar orientation. *A*: 3D reconstruction of an MSO filled with biocytin during patch-clamp recording. *B*: same neuron as in *A* showing cell body and dendrites in green and the axon in red oriented dorsally and medially to its original position in the slice. *C*: MAP2-stained MSO neurons with VGLUT1-labeled dendrites visible toward the lateral (*right*) but not the medial (*left*) side. Medial dendrites seem to be cut perpendicular. *D*: cleared neurons from image shown in *C* show the medial dendrites in a <180° angle compared with the lateral dendrites. *E*: examples of a MAP2-labeled (P60) and a biocytin-filled (P15) MSO neuron show excitatory (VGLUT1) and inhibitory (GLYT2) inputs at the soma as well as the dendrites.

Bipolar morphology and mediolateral dendritic orientation are hallmarks of MSO neurons in several species (Magnusson et al. 2005; Ollo and Schwartz 1979; Rautenberg et al. 2009; Scott et al. 2005; Smith et al. 2000). To study the morphology of MSO neurons in mouse, cells were filled with biocytin during patch-clamp recording and then subjected to immunohistochemical labeling and 3D reconstruction. Mouse MSO neurons indeed have a bipolar shape, with two main dendrites leaving the cell body in opposite directions (Fig. 7*A*). However, orienting the neurons to their original position in the brain slice revealed a caudally oriented bend in the medial dendrites (Fig. 7, *B–D*). This asymmetry is particularly obvious when labeling the excitatory synaptic terminals in coronal brain sections. Starting from the cell bodies, the lateral dendrites extend out laterally and coplanar with the section, while the medial dendrites extend perpendicularly to the cell body and lateral dendrite. During the sectioning of the brain these medial dendrites are frequently cut so that their diameter (red MAP2 labeling in Fig. 7*C*) is surrounded by VGLUT1-positive inputs. Digitally extracting neurons in the same image shows the dendritic orientation asymmetry of the MSO cells more clearly (Fig. 7*D*), where medially projecting dendrites are truncated in the plane of section. Immunolabeling for markers of glutamatergic and glycinergic terminals on a single-cell level shows a mixture of excitatory and inhibitory inputs at both the soma and the dendrites (Fig. 7*E*). To strengthen this finding, we analyzed the VGLUT1 and GlyT2 distribution by plotting the staining intensity in arbitrary units against the medial-lateral position of the MSO neurons. The two resulting intensity distributions were subjected to Gaussian fits from which half-widths were extracted. A half-width ratio near 1 between VGLUT1 and GlyT2 fits would indicate a similar spatial organization of excitatory and inhibitory inputs, while ratios >1 would suggest that the excitatory inputs are spread out onto the dendrites while the inhibitory inputs are concentrated at the soma. The mean ratio of 0.89 ± 0.07 (*n* = 6) supports an equal distribution of excitatory and inhibitory inputs on soma and dendrites of MSO neurons in mice. This pattern contrasts with the arrangement of somatic inhibitory inputs and dendritic excitatory inputs observed for MSO in adult cats and gerbils (Clark 1969; Kapfer et al. 2002).

#### Binaural input to mouse MSO neurons provides an advantage for temporal processing.

**Fig. 8.**
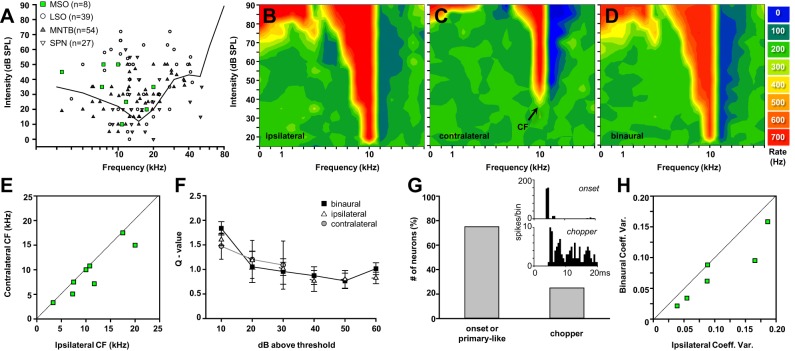
Spectral and temporal properties of mouse MSO neurons in vivo. *A*: frequency-threshold plot relating MSO neurons to other SOC neurons in mice. Solid line represents the psychophysically measured hearing threshold of the mouse (Ehret 1983). *B*: ipsilateral frequency-intensity tuning curve showing both excitation (red) and inhibition (blue) relative to the level of spontaneous activity (green). *C*: contralateral frequency-intensity tuning curve. Arrow indicates the characteristic frequency (CF) of the neuron. *D*: binaural frequency-intensity tuning curve of neuron shown in *B* and *C*. *E*: correlation between the CF of ipsilateral and contralateral evoked responses. *F*: Q values (bandwidth above threshold/CF) against dB above threshold for ipsilateral, contralateral, and binaural responses. Contralateral thresholds tended to be higher, so only Q values up to Q30 were calculated. *G*: distribution of temporal patterns of mouse MSO neurons. *Inset*: examples for onset and chopper patterns at CF/80 dB SPL (0.5-ms bin width). *H*: correlation between ipsilateral and binaural coefficient of variation for the first spike latencies at CF.

Because of the small size of the MSO in mice, targeting the MSO during in vivo recordings is difficult and has a low yield. However, we were able to allocate a small sample of 8 of 128 neurons recorded in the mouse SOC to the MSO on the basis of recording site reconstructions and physiological characteristics (Fig. 8*A*). Frequency tuning curves recorded during ipsilateral, contralateral, and binaural stimulation showed inhibitory sidebands suggesting a mixed input of excitation and inhibition from both ears (EI/EI). The characteristic frequencies (CFs) ranged from 3.3 kHz to 20 kHz, and although contralateral CFs (9.5 ± 1.7 kHz; *n* = 8) had a slightly lower mean than ispilateral CFs (11.0 ± 1.9 kHz; *n* = 8), this was not significant (Wilcoxon signed-rank test: *P* = 0.25; Fig. 8, *B–E*). To test whether the binaural stimulation caused a sharpening in the tuning function compared with unilateral stimulation, Q values were calculated for 10–60 dB above threshold (Fig. 8*F*). No significant differences were found between monaural and binaural Q values (ANOVA: *P* = 0.269; *n* = 4). Temporal response patterns were plotted as peristimulus time histograms and were classified as either onset/primary-like responses (6/8; Fig. 8*G*, *inset*) or chopper responses (2/8; Fig. 8*G*, *inset*) depending on whether their interspike interval histogram followed a Poisson distribution or showed multiple peaks, respectively. These results confirm MSO response patterns observed in other, high-frequency-hearing species (Grothe et al. 1997; Guinan et al. 1972a, 1972b; Pecka et al. 2008). The peristimulus time histograms of the recorded neurons at their respective CF and 70–80 dB SPL were used to measure the first spike latencies and calculate the coefficient of variation (CV) of these responses. Plotting the sole ipsilateral CV (0.103 ± 0.024) against the binaural CV showed a significant improvement of temporal precision in the binaural response (0.076 ± 0.020; paired *t*-test: *P* = 0.036; *n* = 6). With a CV of 0.076 the mouse MSO shows a remarkable precision in latency that is comparable to the values reported for the MSO of the gerbil (5% = 0.05; R oberts et al. 2014).

## DISCUSSION

We have employed patch-clamp recordings in brain slices, single-unit recordings in vivo, immunohistochemistry, and confocal imaging to provide the first comprehensive characterization of MSO neurons in mouse. Our results demonstrate that the location, cell morphology, input projections, and biophysical properties of mouse MSO neurons are consistent with those observed in the MSO of low-frequency-hearing species. This study focuses on describing common features of MSO neurons and may obscure some interspecies differences. However, elucidating the fundamental properties of mouse MSO anatomy and physiology was a necessary first step to validate the mouse MSO as a model system. Detailing the exceptional features of this structure will be a focus of future studies. For example, the mouse MSO deviates from the classical MSO structure with respect to both the arrangement of inhibitory inputs and the overall arrangement of cell bodies and dendrites, similar to the MSO in other small mammals like bats or opossums.

### 

#### Identification of MSO within mouse SOC.

The MSO in mouse is clearly smaller and less organized than in many other mammals and is therefore more difficult to distinguish from the surrounding nuclei. Low-magnification images obtained for each recorded neuron during patch-clamp experiments allowed clear exclusion of MNTB and LSO neurons. SPN or ventral nucleus of the trapezoid body (VNTB) neurons (which are located just dorsal or ventral to the MSO, respectively) are possible sources of false positive identifications; however, the physiological responses of these neurons were distinct from those of MSO neurons. Mouse MSO neurons respond to sustained depolarizing current injections with a single, onset action potential, consistent with reported responses from gerbil and guinea pig (Baumann et al. 2013; Couchman et al. 2010; Scott et al. 2005; Smith 1995). SPN and VNTB neurons typically fire trains of large-amplitude action potentials in response to current injections at amplitudes only slightly above threshold (SPN: Felix et al. 2013; Kopp-Scheinpflug et al. 2015; Yassin et al. 2014; VNTB: Tong et al. 2013). SPN neurons can be distinguished by firing rebound bursts after hyperpolarizing current injection, a phenomenon rarely observed in MSO neurons (Felix et al. 2011, 2013; Kopp-Scheinpflug et al. 2011, 2015; Yassin et al. 2014). VNTB neurons typically have high *R*_input_ (>300 MΩ) and exhibit a large voltage drop with the small current steps (Sinclair JL, unpublished observations). In contrast, mouse MSO neurons shared a low *R*_input_ (<20 MΩ by P21) similar to age-matched gerbils (∼10 MΩ; Lehnert et al. 2014; Magnusson et al. 2005).

In most mammals (Grothe 1994), MSO neurons send their projection mainly to the ipsilateral inferior colliculus (Roth et al. 1978; Schofield and Cant 1991) and few collaterals to the contralateral inferior colliculus (Brunso-Bechtold et al. 1981). Fluoro-Gold injections into the inferior colliculus retrogradely labeled a distinct population of neurons in the MSO region and only on the ipsilateral side (Fig. 1*F*). Further confirmation of MSO identity derives from immunohistochemical and biophysical evidence of prominent glutamatergic and glycinergic synaptic input to neurons in the putative mouse MSO.

#### Voltage-gated conductances in mouse MSO.

MSO firing properties are largely determined by a unique expression pattern of several voltage-gated channels that control membrane excitability. Mouse MSO neurons exhibit biophysical characteristics similar to other species including high expression of K_v_1 and K_v_3 potassium channels and the nonspecific cation channel *I*_H_.

Strong low-voltage-activated K_v_1 currents are observed in neurons throughout the temporal pathway of the auditory circuit in birds and mammals (Brew et al. 2003, 2007; Cao and Oertel 2011; Fukui and Ohmori 2004; Gazula et al. 2010; Johnston et al. 2010; Klug and Trussell 2006; Manis and Marx 1991; Mathews et al. 2010; Rosenberger et al. 2003). The reduced excitability suppresses action potential responses to temporally distributed inputs and limits dendritic filtering (Lehnert et al. 2014; Mathews et al. 2010; Scott et al. 2005). The difference in amplitudes of low-voltage-activated K_v_1-mediated currents in response to perithreshold depolarizations is between ∼1 nA in the mouse (present study) and ∼4 nA in the age-matched gerbil (Scott et al. 2005). This difference in magnitude is likely to be attributed to the general neuronal size difference between the two species.

Strong K_v_1-mediated currents are often complemented by high expression of hyperpolarization-activated cyclic nucleotide modulated currents (*I*_H_) (Cao and Oertel 2011; Golding and Oertel 2012; Khurana et al. 2011). These channels set the resting membrane potential and reduce *R*_input_, allowing neurons to rapidly recover from depolarization events. *I*_H_ currents with fast activation kinetics mediated by the HCN1 subunits have been described (Baumann et al. 2013; Khurana et al. 2012; Koch et al. 2004; Kopp-Scheinpflug et al. 2015), similar to the analogous neurons in the avian nucleus laminaris (Yamada et al. 2005). Our observations in mouse MSO neurons include prominent depolarizing voltage sag responses to sustained hyperpolarizing current injections, indicative of strong HCN channel expression.

The high-voltage-activated K_v_3 currents contribute to the rapid repolarization of the membrane during action potentials (Johnston et al. 2010), making them crucial for the high-frequency firing observed in the temporal processing circuitry of the auditory system. TEA broadened the action potential waveform without changing overall membrane responses to sustained current injection (e.g., firing rate), consistent with high K_v_3 channel expression. Taken together, this is compelling evidence that these are MSO neurons and that they are functionally specialized for processing temporal features of stimuli.

#### Synaptic inputs.

EPSCs are remarkably fast in MSO of rats and gerbil, with decay time constants as rapid as 0.2–1 ms (Chirila et al. 2007; Couchman et al. 2010; Fischl et al. 2012; Jercog et al. 2010; Smith 1995). Spontaneous and evoked EPSC responses from the mouse MSO were consistent with these values, with an average decay time constant of 0.83 ± 0.1 ms.

IPSCs in gerbil and rat MSO are predominantly glycine mediated at ages >P14 and are also remarkably rapid, with ∼1.3–3 ms decay time constants depending on species and age (Chirila et al. 2007; Couchman et al. 2010; Fischl et al. 2012; Magnusson et al. 2005; Roberts et al. 2014; Smith et al. 2000). Mouse MSO eIPSCs and spontaneous IPSCs fell within this range, with decay time constant of the maximum eIPSCs being 1.68 ± 0.18 ms (*n* = 12). For both IPSCs and EPSCs, kinetic measures of spontaneous and evoked PSCs were highly correlated, suggesting that evoked vesicle release is highly synchronous.

MSO neurons have a distinct bipolar morphology, with dendrites that extend in the mediolateral plane, each receiving input derived from one ear (Russell and Moore 1999; Stotler 1953). Generally, immature MSO neurons are more elaborately branched with thin dendrites. During development the branched morphology coalesces down to relatively branchless, large-diameter dendrites by about P20 (Chirila et al. 2007; Rautenberg et al. 2009). This morphology is present in cats, bats, rats, guinea pigs, gerbils, and humans (cats: Lindsey 1975; Stotler 1953; bats: Grothe and Park 2000; rats: Smith et al. 2000; guinea pigs: Smith 1995; gerbils: Chirila et al. 2007; Couchman et al. 2010; Franken et al. 2015; Magnusson et al. 2005; Scott et al. 2005; humans: Kulesza 2007). In the present study, MSO neurons, either stained with MAP2 or filled with biocytin during patch recordings, revealed typical MSO-like bipolar morphology (Fig. 7*E*). However, qualitatively two anatomical features of mouse MSO neurons were distinct from other species. First, even in older tissue, dendrites were thin diameter and branched, resembling immature gerbil MSO neurons. Second, the orientation of MSO dendrites was neither orderly among cells, a feature also described for bats and opossums (Grothe 1994; Kapfer et al. 2002), nor planar for individual neurons. Instead, lateral MSO dendrites projected laterally, but medial dendrites tended to turn caudally, a feature not described before.

Similar to the MSO in bats and *Monodelphis*, the glycinergic inputs to the mouse MSO seem not to be restricted to the cell somata. In contrast, the glycinergic inhibitory inputs to MSO in cats and gerbils are restricted to the cell somata (Clark 1969; Kapfer et al. 2002), a feature that only develops upon experience after hearing onset (Kapfer et al. 2002) and correlates with ITD tuning as measured at higher levels of the ascending auditory system (Seidl and Grothe 2005).

#### Function of the MSO in mouse.

The present study was conducted in order to compare and contrast the known features of MSO neurons from several species with the mouse MSO for the purpose of providing a baseline for future investigation. We did not explicitly conduct tests to assay auditory function beyond our limited sample of in vivo recordings, where data from MSO cells were obtained as part of a broader survey of physiological responses in the mouse SOC and constituted just 6% (8/128) of our sampled neurons. Binaural selectivity was not a primary goal of this set of experiments. Nevertheless, in these cases data were sufficient to demonstrate physiological response type and neuron location consistent with the MSO. A directed and sustained effort will be necessary to target MSO neurons explicitly and to hold these neurons long enough to yield comprehensive binaural stimulus response profiles. This is a worthy goal but beyond the scope of this initial study. However, some functional roles may be speculatively proposed based on previous studies from other high-frequency-hearing species. It is clear that the MSO, which is known to exhibit ITD selective responses to tones or envelopes in several species, is unlikely to do so in the mouse, which is predicted to not receive useful ITD cues for sound localization (Grothe and Park 1998; Harper and McAlpine 2004).

Studies in several bat species reveal anatomical and electrophysiological MSO neural properties shared with those of low-frequency-hearing mammals (Grothe et al. 1992, 1994, 1997; Grothe and Neuweiler 2000). However, their MSO also deviates in some aspects from the classical MSO: the cell bodies of MSO neurons are not orderly aligned in one plane, and the inhibitory inputs are, as in small opossums, not restricted to the cell body and proximal dendrites as, for instance, in cats or gerbils (Kapfer et al. 2002). To date there have been no comprehensive cellular biophysical studies describing MSO neurons in small and exclusively high-frequency-hearing mammals such as mice and bats. Thus it is unknown whether MSO neurons in these species share the same distinct biophysical properties that have been described in detail for MSO neurons in gerbils and rats, two small mammals with low frequency hearing. A clear example of pure nonspatial temporal coding from the MSO is found in the mustached bat *Pteronotus parnellii* (Grothe 1994). The mustached bat MSO receives monaural ipsilaterally derived excitation and inhibition. These MSO neurons exhibit selectivity for amplitude modulation rates from sinusoidally modulated stimuli. This envelope selectivity is disrupted when glycinergic input is blocked, suggesting a role for stimulus-locked inhibitory input. In another exclusively high-frequency-hearing bat species, the Mexican free-tailed bat, *Tadarida brasiliensis mexicana*, the MSO is binaural. These MSO cells time-lock to the envelope of tones presented at the neuron's best frequency and exhibit ITD selectivity when these modulated tones are presented binaurally. However, the envelope sensitivity appears too low for microsecond ITD processing and therefore has been interpreted as epiphenomenal and without behavioral relevance for direct sound localization (Grothe and Park 1998). It can only be speculated that the binaural nature of temporal processing in the mouse and bat MSO may serve processing of spatial cues in complex situations with reverberations or additional masking sound sources (Grothe and Neuweiler 2000) that create ILDs or ITDs beyond ITDs occurring from direct sounds alone (Fitzpatrick et al. 2002; Grothe and Park 1998; Meffin and Grothe 2009).

#### Potential insights from genetic manipulation of mouse MSO circuit.

Genetic manipulation of the mouse genome is a powerful tool that has been exploited to test innumerable hypotheses over the last several decades. Given the similarity of the physiology of the mouse MSO to other species, the mouse could be used as a genetic model for investigating many features of the MSO where genetic manipulation is advantageous.

For example, well-annotated and published sequence expression data from the mouse show strong expression of the *Ether-à-go-go*-related gene (ERG, K_v_11) channel in the MSO (Allen Institute 2015; Lein et al. 2007). During the processing of auditory stimuli, ERG channels assist K_v_1 channels in determining spike threshold and may play a role in resetting the interspike potential in a stimulus train (Hardman and Forsythe 2009), an important trait for phase-locking behavior in MSO neurons. Since systemic application of ERG channel blockers in vivo is prone to induce cardiac arrhythmias like the LQT syndrome (Vandenberg et al. 2012), genetic manipulation of ERG channels might be a favorable approach to investigate their role in the MSO.

Genetic manipulation of the mouse MSO circuit could also be useful when studying ion channel groups for which highly selective pharmacological manipulations are lacking. The biophysical role of specific HCN1-4 subunits in the MSO has been difficult to parse physiologically. Our study suggests that the mouse provides a model for which investigation of these properties is now feasible with genetic tools. The K_v_3 group is another example of a family of channels that lack specific antagonists (Rudy and McBain 2001). In a human study, a familial mutation of the K_v_3.3 channel subtype resulted in decreased sensitivity to ITDs and ILDs (Middlebrooks et al. 2013). Again, the mouse model provides a platform to extend these investigations to the cell physiology level.

#### Summary.

The presence of MSO cells in mice that physiologically and anatomically resemble not all but many key features of those in cat, guinea pig, rat, and gerbil provides a new animal model to study questions about the MSO circuit that require genetic manipulation available for mice that is not yet available for larger-headed animals. The biophysical properties of the mouse MSO neurons exhibit many characteristics associated with encoding temporal features of acoustic input as shown in vivo for other mammals limited to high frequency hearing (e.g., envelope coding). This may contribute to localization processing in complex conditions and to signal detection in noise. Future in vivo studies of mouse MSO neurons with more complex stimuli will be required to gain insight into the particular functional contributions of these neurons. Overall, the data presented here strongly suggest that mouse MSO neurons allow investigation of cellular MSO features using the powerful genetic tools available in the mouse model.

## GRANTS

This research was funded by Deutsche Forschungsgemeinschaft (DFG) KO2207/3-1 and SFB870-A10 (C. Kopp-Scheinpflug), the Alexander von Humboldt Foundation (R. M. Burger return fellowship), and Medical Research Council (MRC) K005170 (I. D. Forsythe).

## DISCLOSURES

No conflicts of interest, financial or otherwise, are declared by the author(s).

## AUTHOR CONTRIBUTIONS

M.J.F., R.M.B., M.S.-P., O.A., J.L.S., and C.K.-S. performed experiments; M.J.F., O.A., and C.K.-S. analyzed data; M.J.F., R.M.B., M.S.-P., O.A., J.L.S., and C.K.-S. prepared figures; M.J.F., R.M.B., O.A., J.L.S., B.G., I.D.F., and C.K.-S. edited and revised manuscript; M.J.F., R.M.B., M.S.-P., O.A., J.L.S., B.G., I.D.F., and C.K.-S. approved final version of manuscript; R.M.B. and C.K.-S. conceived and designed research; R.M.B., O.A., B.G., I.D.F., and C.K.-S. interpreted results of experiments; R.M.B. and C.K.-S. drafted manuscript.
